# P-770. Blood Culture Utilization and Bacteremia Rates in Staphylococcus aureus Bacteriuria: A Retrospective Single-Center Study

**DOI:** 10.1093/ofid/ofaf695.981

**Published:** 2026-01-11

**Authors:** Ian Gibson, YoungYoon Ham, Kendall J Tucker, HaYoung Ryu

**Affiliations:** Oregon Health & Science University, Portland, OR; Oregon Health & Science University, Portland, OR; Oregon State University College of Pharmacy, Portland, Oregon; Oregon Health & Science University Hospital and Clinics, Portland, Oregon

## Abstract

**Background:**

*Staphylococcus aureus* bacteriuria (SABU) has been associated with invasive disease, yet optimal management remains unclear. Blood cultures are generally recommended, but adherence to this practice appears inconsistent. We evaluated SABU management and outcomes at a quaternary care center.

**Table 1:**
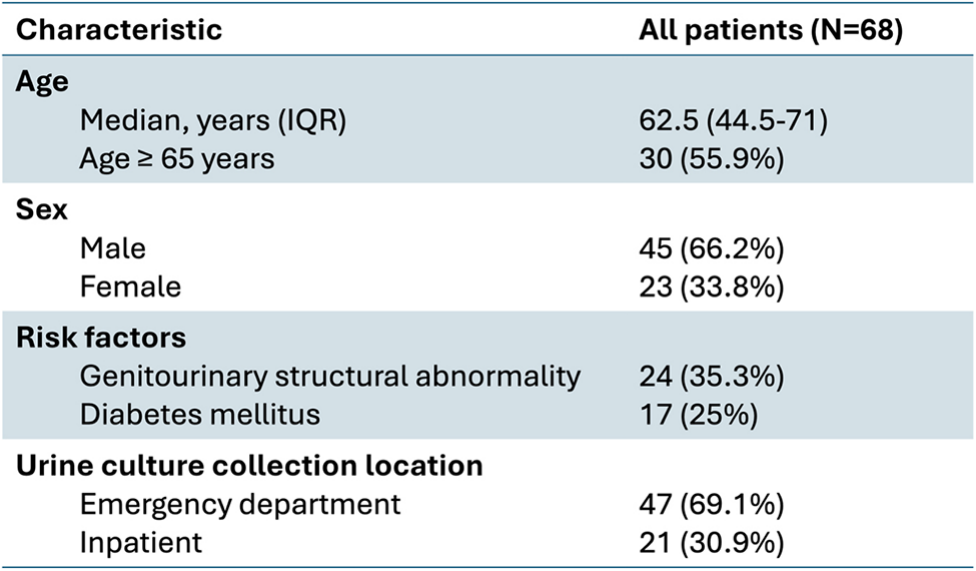
Characteristics of the Patient Population

**Table 2:**
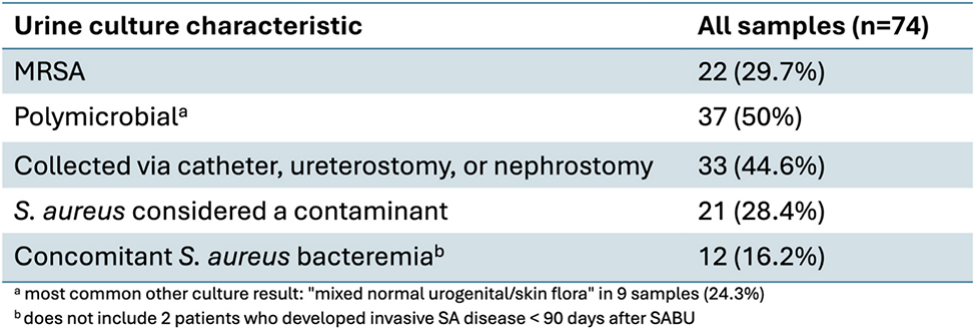
Characteristics of the Collected Urine Cultures

**Methods:**

We conducted a retrospective review of all urine cultures positive for *S. aureus* (SA) collected in the hospital, including the emergency department, between 08/20/2023 and 08/20/2024 using an electronic clinical surveillance system. Data included temperature, white blood cell (WBC) count, urine culture details, urinary tract infection (UTI) symptoms, antibiotic use, presence of other infections, and development of invasive SA disease. The primary outcome was the proportion of SABU cases with blood cultures obtained and associated variables. Data were analyzed using descriptive statistics.

**Figure:**
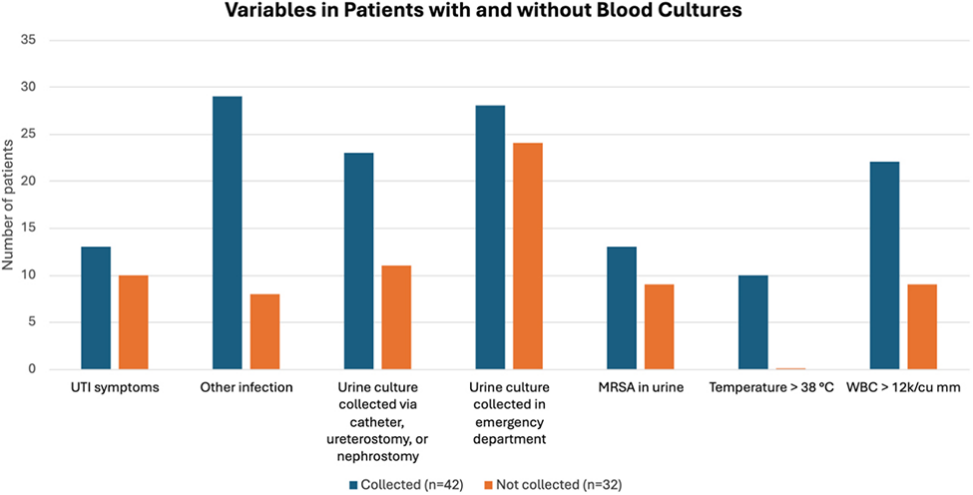
Comparison of Variables in Patients with and without Blood Cultures

**Results:**

Seventy-four urine cultures from 68 patients were positive for SA, with significant bacteriuria (≥ 10⁵ CFU/mL) in 45%; 30% were methicillin-resistant *S. aureus* (MRSA). Seven patients (10%) had invasive SA infections (bacteremia, osteomyelitis, or septic arthritis) at the time of urine culture. Blood cultures were obtained in 57% (n=42), with SA bacteremia in 29% (n=12). Among those with blood cultures, only 12% had both fever (≥ 38 °C) and leukocytosis (WBC ≥ 12x10³/μL). In patients without blood cultures, none were febrile, but 21% had leukocytosis alone. Blood cultures were more likely with another infection (78%) or urine collected via catheter, ureterostomy, or nephrostomy (61%). Antibiotics for UTI were used in 41% of cases. Notably, 43% of antibiotic recipients had no reported UTI symptoms. SA was considered a contaminant in 28% of urine cultures. Two patients developed invasive SA infections within 90 days; neither had blood cultures drawn or signs of systemic infection at the time of SABU.

**Conclusion:**

Blood culture collection in SABU was inconsistent and often guided by concurrent infections or urine collection method rather than SABU itself. Given the observed bacteremia rate and the potential for progression to invasive disease, standardized evaluation including blood cultures may improve early detection of invasive disease and guide appropriate management.

**Disclosures:**

Kendall J. Tucker, PharmD, MS, Merck: Grant/Research Support

